# Puerperal Infection1The President’s valedictory address before the Buffalo Academy of Medicine, June 12, 1900.

**Published:** 1900-08

**Authors:** Matthew D. Mann

**Affiliations:** Buffalo, N. Y., 37 Allen Street


// BUFFALO MEDICAL JOURNAL.

VOL. XL.—LVI. AUGUST, 1900. No. 1.

### Original Communications.

### PUERPERAL INFECTION.[1]

BY MATTHEW D. MANN, A. M., M. D., Buffalo, N. Y.

IN LAYING down my office, I look back upon the time of my incumbency as being one of the most important periods in the history of the Academy. Three events have marked the year, all of which are steps in progress, and must have a considerable influence in the history of our Association.

My immediate predecessor, Dr. Park, in his valedictory address suggested that the publication of the Transactions would be of great use to the Academy and to the profession. As I had long held this opinion, I determined at once to try to have the idea carried out. Dr. Potter, of the BUFFALO MEDICAL JOURNAL, was very willing to accept the Transactions, and I found the secretaries of the various sections, when the matter was clearly put to them, quite willing to get up reports of the meetings for publication. How well they have done their work, you all know. They certainly have accomplished very creditable results, and the Academy owes them a debt of gratitude. Personally, I am very deeply indebted to them all for having so ably followed out my suggestions. The plan having been once inaugurated, I do not believe it will be likely to be given up. It cannot but add to the strength of the Academy, and ought to make its discussions of more interest and importance, and to spread its influence widely throughout the profession.

The second event of importance was the establishment of a new section devoted to diseases of the eye, ear, nose and throat. The idea of this was not new to me, and when Dr. Bennett brought it by

1. The President's valedictory address before the Buffalo Academy of Medicine, June 12, 1900.

resolution before the Council, I was very glad to throw my influence in its favor. It is directly in the line of growth and development, and I feel sure that the new section will increase the interest in the departments to which it is devoted and add materially to the usefulness of the Academy.

The rooms which we so long occupied on Main Street were never very desirable, and at times during the last year they have been crowded to their utmost.

The necessity for a better meeting place for the Academy has long been felt, and the trustees have now provided a most excellent place, the one in which we now are. The offer of the University of Buffalo, to give a room, free of expense to the Academy, while it had some advantages, did not seem to meet with general approval. The hall put at the disposal of the Academy is not well arranged for such meetings, and I think the Academy did very wisely in refusing the offer.

The plan of dropping those of the Fellows who were several years behind-hand in the payment of their dues, was according to the constitution, and has worked well in two ways In the first place, it has added materially to the income of the Academy; and, secondly, it has cleared the rolls of the names of those who have ceased to be active and interested Fellows.

I am sorry that the increase in membership during the last year has not been greater. A large number have been asked to join, but have refused. The membership should include all the active members of the profession in Buffalo and its suburbs.

The character of the papers which have been read during this year is certainly up to the standard of previous years. Some of the out-of-town papers have been of great interest and have drawn large crowds. It does seem to me, however, that many of the papers by our home Fellows have been quite as good and quite as deserving of a good turn-out as those which were read by outsiders. We certainly should not neglect our own Fellows when they prepare themselves to entertain us. I have been quite ashamed at the attendance on some evenings, and proud of the papers which have been read, and wished very heartily that the writers had been encouraged and complimented by a larger audience. I sincerely trust that interest in the Academy will increase, and that it will continue to prosper, until it becomes one of the leading medical societies of the country. To my successor and his assistants, I leave the task of carrying on the good work, and of introducing new features, feeling confident that they will have a support equal to that which you have so generously afforded me. In the name of the Academy, I wish to thank the various officers who, during the last year, have carried on its affairs so successfully.

. . . . . . . . . . . .

I will now pass from the consideration of the Academy matters, and ask your attention to one which is of great importance to every general practitioner. I have nothing new or original to offer, but have striven to collect the latest ideas regarding puerperal infection. I know that many general practitioners are too busy with their round of work to allow of careful study of all the newest books and journals, and, while I realise that you are all familiar in a general way with the advances which have been made in our knowledge of puerperal infection, still I think it will be good for us if I gather together in concrete form the latest ideas, together with what seem in my judgment to be the best rules of practice.

Up to very recent times—in fact, within the lifetime of many here present—the whole subject of puerperal disease was wrapped in deep gloom and obscurity. The first ray of light came from the writings of Oliver Wendell Holmes, who, in 1843, entered an earnest plea for the acceptance of the doctrine of the contagiousness of puerperal fever. A few years later Ignatius Semmelweis, then an assistant in the General Hospital in Vienna, showed the identity of puerperal and surgical fevers, and that puerperal sepsis was conveyed from the postmortem room by the hands of the examining students. Both Holmes and Semmelweis were met by the most determined opposition. Satire and ridicule, invective and abuse, were heaped upon them; but, in a very short time, both were shown to be right, and their ideas are now the commonly received theories of the profession.

I wish I had time to give you a brief review of Semmelweis's history. It is exceedingly interesting, and only shows how hard it is for the human mind, unless it is filled with the true scientific spirit, to accept new ideas. I do not think anyone today could be treated as Semmelweis was for the advocacy of a new pathological doctrine. Poor Semmelweis finally wore himself out and fretted himself into insanity. He could not stand the ridicule and opposition to which he was subjected. Curiously enough, he died of a septic wound received during an operation, just before he was committed to an asylum.

As Hirst says, "It is to be hoped that the medical world of today, and of the future, can never be deaf and blind to such an appeal as that of Holmes, or to such a demonstration as that of Semmelweis."

Later came the discoveries of Tyndall, Pasteur and Lister. The idea of a distinct puerperal fever was then given up, and we now recognise the processes which are grouped under this heading as being practically the same as those which are found elsewhere and under other conditions. Following labor, the genital canal very closely resembles a wound made by a surgical operation. The frequently occurring lacerations of the cervix and perineum, when they are present, make the analogy still closer. The study of pathology has shown that the diseased processes affecting surgical wounds and the puerperal genital tract are precisely similar, only modified in a slight degree by the peculiar conditions in each case.

So now we understand puerperal fever to be really puerperal septicemia, and to be due to the entrance into the system of certain bacteria which are known to have definite pathogenic properties.

The bacteria which have been found to be the causes of puerperal fever are the streptococcus, the staphylococcus, the bacillus coli communis, and the gonococcus. Besides these, a considerable number of bacteria have been found in the organs and secretions of women sick with puerperal fever. The Klebs-Löffler bacillus is, perhaps, the most important. I will not, however, weary you with a long list of doubtful forms.

The main question for us, as practical obstetricians, is, how do these bacteria get in? Are any of them found normally in the genital passages, or do they always get in afresh from the outside? If so, how can their entrance be prevented?

In the earlier stages of bacteriological study, it was supposed that the vagina was a perfect hotbed of bacteria; hence arose the theory of autoinfection. Later and more careful investigations have shown that not only is the healthy uterus absolutely free from pathogenic bacteria, but that the vagina, in a state of health, is equally so, and that nature has provided an elaborate system of defence against the entrance of germs into this canal.

The first argument against autoinfection is the fact, just alluded to, that the vagina is sterile. Döderlein has shown that there exists within the vagina an anërobic bacillus. This bacillus secretes lactic acid, which is antagonistic to staphylococci, and which, within certain limits, destroys them. Experiments have been made of infecting the vagina of a virgin with staphylococcus cultures in large quantities. Within four days, the vagina was found to be entirely free of the injected germs. Döderlein further supported his theory in the following facts:

First, all pathological secretions containing germs are weakly acid or alkaline.

Second, in puerperal women the vaginal bacillus disappears, and in its place are found many kinds of saprophytes, the lochial discharge being alkaline.

Third, when the lochia ceases, the saprophytes disappear, the vaginal bacillus reappears, and the vaginal secretion becomes intensely acid.

Döderlein's conclusions have been challenged, and many further experiments made. J. Whitridge Williams, after a series of very careful observations and a study of the work of other investigators, has shown conclusively that Döderlein is correct, and has pointed out their errors to those who have differed from him. Other experimenters have coincided with Döderlein in their results. Krönig, in 200 examinations, found no pathogenic microörganisms in the vagina. He attributes the sterility of the vagina to the outward flow of the vaginal secretions. Menge thinks that the germicidal action is aided by the anatomical elements of the vagina, by a leucocytosis, due to irritation in the vagina, and a consequent phagocytosis. There are numerous other observations and theories in this interesting subject, but I think we may sum it all up by saying, that the healthy vagina and uterus, either in pregnant or nonpregnant women, are always sterile, and that autoinfection from this source is not possible.

While this may be true of the uterus and vagina, it certainly is not true of the vulva. In an address delivered in 1899, Dr. J. Clifton Edgar has given the results of very careful bacteriological observations made upon the vulvæ of pregnant women. Of thirty cases examined, he sums up the results as follows: The staphylococcus albus was found eight times; s. aureus, three times; streptococcus, once; negative or sterile, nineteen cases. Coli communis was not found at all.

These findings show pyogenic bacteria of the vulvar orifice present in 40 per cent. of the cases examined. These observations are very important; and, while they do not show that there can be an autoinfection—the vagina being imposed as an impassable barrier between the uterus and the vulva—they do show that a source for infection is very close at hand in many cases, and, while this source is on the surface of the body, it is none the less important and is so situated that it can be very readily carried into the deeper tissues. We must remember also that after labor the acid secretion of the vagina disappears, and thus the growth of germs which may have been introduced from the outside is favored. This alkalinity of the lochia is doubtless due to the large amount of blood serum which is poured out, and to the absence of the bacillus vaginæ, which seems to be destroyed at the time of labor.

There are two other arguments which are used against autoinfection: one is the result of douching preliminary to labor. If there are germs in the vagina and they produce the morbidity so common after labor, then preliminary douching with antiseptics should give better results than in cases where the douching is not used. There is an immense amount of clinical evidence pro and con, but the weight of authority is all against the douche. For instance, Leopold used the douche in 5,784 cases; 82.4 per cent. were free from fever during the puerperium. He then treated 4,088 without the douche, and found that 92.2 per cent. were free from fever. Similar statistics might be quoted in much larger numbers, and mostly with the same results.

Still another argument against autoinfection is the results obtained in certain hospitals where great attention is paid to cleanliness and the careful conduct of the labors. If such a thing as autoinfection were possible, then the results obtained in these hospitals would be impossible because a certain proportion of the cases would be infected notwithstanding the greatest care. The following statistics are worthy of consideration on this point: Sloan Maternity, no mortality from sepsis since its foundation. New York Maternity with 1321 cases, no deaths from sepsis. New York Post-Graduate, 850 cases with one death. New York Polyclinic, 450 cases, no deaths. University Hospital, Philadelphia, no deaths in six years. Preston Retreat, 480 cases, no deaths. Boston Lying-in Hospital, 2,194 cases, 1 death.

Having thus disposed of the question of autoinfection, we may consider still further how infection is introduced from without. It must be remembered that infection may be present in the genital tract a long time before labor; then it may spread postpartum, giving an appearance of autoinfection. But this is not a true autoinfection, but rather an external infection which has become latent. What, then, are these latent infections which so antedate labor and cause an extention of infection during the puerperium? Unquestionably, the most common is gonorrhea.

Noeggerath, in 1876, said that 80 per cent. of males had chronic gonorrhea; that 90 per cent. of these were never cured, and could infect. Therefore, of 100 women whose husbands had had gonorrhea, scarcely 10 were well, and the majority were sterile. While there was much truth in this, it was a great deal too strong, and Noeggerath later admitted it.

Sänger agreed with Noeggerath. He claimed that one-ninth of all the disease of the female genitals were due to gonorrhea; that 50 per cent. of these cases had diseases of the tubes and peritoneum, the result of an ascending infection during the puerperium. Sänger later reduced his percentage; but, to show how often it occurred in the puerperium, he examined the pregnant women in the Leipsic Clinic, and found that 26 per cent. of them had gonorrhea. Oppenheimer, in the Heidelberg Clinic, found 27 per cent. of pregnant women had gonorrhea, and 40 per cent. of the children had ophthalmia neonatorum, due to the gonococcus. Lomer, in Berlin, found 28 per cent. of pregnant women to have gonorrhea. Notwithstanding the great numbers of infected women, the mortality in their confinements was small, showing that this form of infection is rarely fatal.

Sänger holds that in these cases there are two processes—either a latent gonorrheal infection, which ascends during the puerperium, and which is caused by a pure culture of the gonococcus; or, secondly, a septic infection added to a previously existing gonorrheal infection. Clinically, he finds that these two processes differ very materially, particularly in the date at which they occur. The pure gonorrheal infection usually shows itself in the third or fourth week, as it takes considerable time to spread; whereas the mixed infection develops soon after labor. It has been shown that the gonococcus can penetrate pavement epithelium; that it is a true pus-producing germ, and can produce peritonitis, and has been found in the peritoneal exudates in pure culture. Pure cultures have also been found in pus-tubes and ovarian abscesses. In ninety cases of pus-tubes where bacteriological examinations were made, 50 per cent. were found to have a pure gonorrheal infection, while in many the gonococcus was present with other germs.

It being admitted that the cervix is the chief seat of gonorrheal infection, and that the pathogenicity of the germs is often of so low a grade that they do not attempt to spread, we can readily understand that the disease would become latent in the cervix and remain there for an indefinite period. This is now generally accepted. After labor the conditions are entirely changed. The lochia afford a good culture medium for the germs, and gradually they spread, until they invade the entire genital tract. They are much milder and slower in their action than are the septic germs, and their spread is usually limited after they reach the peritoneal cavity, by a circumscribed peritonitis. Once they are shut up in a cavity containing pus, they seem to soon die, or at least lose their virulence. While they are milder in their action, the inflammation excited by them, although it may be recovered from more often, tends to become chronic and resist treatment and to recur. This condition is often found in a first pregnancy and results then in the so-called "one-child" sterility.

Having disposed of the question of autoinfection, and explained the cause of latent infection, let us now, for a moment, consider the character of the septic process induced by the different germs. Undoubtedly, one of the commonest results of infection is the so-called sapremia. This is caused by the entrance of saprophytes into the vagina and uterus, and their growth on foreign material which has been left there—such as bits of placenta, membranes and blood clots. These growths are not truly septic in character, do not enter the circulation at all, but produce certain toxins which are absorbed and give rise to various symptoms. While these saprophytes are local in their action, they create a condition which is very apt to be followed by true septic infection, so that, although the condition of sapremia may be quickly and readily recovered from if the septic germs are kept out, should they obtain admission, then a very serious complication arises. Unquestionably, the most virulent germ is the streptococcus, and, unlike the saprophytes, it produces no evidence of decomposition; there is no odor to the discharges, and often very little discharge of any kind. Once introduced into the wounded perineum, cervix or uterine body, it tends to reproduce itself very rapidly, and to travel by the lymphatic route, thus making its way into the deep tissues, into the peritoneum, and inducing general systematic poisoning. The staphylococcus is of a somewhat similar nature, but is not so virulent, and is more readily limited in its action by the walling-off process, either in the tissues or in the peritoneal cavity. The colon bacillus is the least virulent of the three. Sometimes doubt has been thrown upon the nature of the processes induced by this last mentioned germ, but I have recently seen reports of a number of cases of septic infection from which were obtained pure cultures of the colon bacillus.

If, then, puerperal infection is due to the presence of infective bacteria within the genital tract, and if there are in healthy pregnant women no bacteria present in the vagina or cervix uteri, how do the bacteria get in to induce the pathological condition found after labor?

Unquestionably, they may get in from the atmosphere. It has been found that hospital walls, floors and beds may become infected, and that disinfection of these would stop a certain epidemic. Gustav Braun found epidemic influences in Vienna, which he thought were due to fecal matter in a canal which flowed close to the lying-in wards. An epidemic was thought to have been started in the maternity wards at Blackwell's Island, by a liberal application of guano to the lawn around it. So cases have been attributed to the proximity of cess-pools, privies, stables, slaughter-houses, open sewers, to pools of stagnant water, and leaking or untrapped waste pipes.

As means of infection, all of these undoubtedly play a very unimportant part as compared with the fingers of the nurse and doctor. It has been shown by bacteriological investigation, that it is almost impossible to thoroughly sterilise the hands. We must also remember the results of investigation of the condition of the vulva. So we may be sure that in the majority of cases the infection is carried in upon the fingers of the doctor or nurse. How the examining fingers become contaminated makes no difference. The germs may be pushed in from the vulva of the parturient woman; they may come from a septic sore on the doctor's person, or from a case of erysipelas, a chronic ulcer—in fact, any source of infection, either in the dead or living body; and especially dangerous are other cases of puerperal septicemia. The dictum of Holmes, said many years ago, but then doubted, is admitted by all as true today: "The disease known as puerperal fever is so far contagious as to be frequently carried from patient to patient by physicians and nurses."

This is a very serious indictment to bring, and should make everybody attending a puerperal case exceedingly careful as to cleanliness of their hands, persons and clothing.

While there can be no doubt of the truth of the statement here made, that infection is introduced by the examining finger more frequently than in any other way, nevertheless, recent observations go to show that certain germs are present in the uterus after labor in a very considerable proportion of cases, whose introduction is apparently not due to the examining finger. In the May number of *Obstetrics*, Dr. Franz, of the University of Halle, shows that germs are often present in the uterine cavity during the normal puerperium. In eighty-one cases out of 300 this was found to be the case. This contradicts Krönig, who says that the cavity of the puerperal uterus is, under normal circumstances, free from germs; the occurrence of microörganisms in this situation is pathologic.

To be sure, most of the germs reported by Franz were saprophytes, very few of them being the true pathogenic germs. In fifty cases of febrile puerperium, thirty-five of which were mild and fifteen severe, Franz found bacteria present in forty-seven. He adopted a new method, cultivating the germs anërobically. His results show that in 69 per cent. of the mild cases of fever in childbed, saprophytes were present; while streptococci were present in 80 per cent. of the severe cases. He assumes, then, that slight elevations of temperature are caused chiefly by saprophytes, while less often in the mild cases we have streptococci and the other pathogenic organisms.

Further, he argued that, if this situation explains the temperature elevation, still the cavity of the uterus must be germ-free in febrile cases. He, therefore, examined ten such cases, and found saprophytes nine times and staphylococci once. Some of his conclusions were as follows:

1. Slight elevations of temperature during the puerperium are usually caused by saprophytes which gain access to the uterine cavity.

2. The saprophytes themselves do not cause fever. It develops only when the outflow of the bacteria-containing secretion is prevented.

3. The saprophytes which are found in the uterus in cases of slight elevation of temperature are probably identical with the saprophytes of the vagina.

These conclusions are interesting, but we have time only for a brief comment upon them at present. It will be noticed that in nearly all the cases the bacteria present in the endometrium were saprophytes. As these are everywhere present in the air, and even in the vagina, according to some observers, and as the normal preventive conditions are absent following labor—the lochia being alkaline and there being much detritus of tissue and blood clots present in it—we can easily understand how these peculiar germs make their way into the uterus during the puerperium. But as they are not pathogenic in themselves, and as the septic germs have been rarely found, this discovery does not take away the responsibility for the introduction of the pathogenic germs by the hands of the attendants.

Time forbids my going into the question of the pathological conditions induced by the presence of the germs. Sufficient to say, that the trouble usually starts as an endometritis, and that the further progress of the disease depends upon the nature of the causative organism. If these be saprophytes, then the disease will probably be localised in the uterus, and the disease shown by a bad-smelling, fetid discharge. If they be streptococci of the virulent type, the endometritis is of very slight importance, the germs rapidly making their way by the lymphatics into the system at large, or into the connective tissue or peritoneum, setting up, as the case may be, an acute septicemia or peritonitis, or, later, a cellulitis, and this followed by a phlebitis or pyemia.

The question of prophylaxis is the most important one. Undoubtedly, cleanliness is here the key to the situation. If the surroundings of the patient, including the atmosphere, are surgically clean; if the vulva is rendered clean at the beginning of the labor, and kept so during its progress; if the hands and the instruments of the obstetrician are equally carefully attended to, then the danger of infection will be reduced to a minimum. If the vagina is normal, as shown by an acid condition of its secretions, the preliminary douche should be avoided. In normal cases, vaginal douches, both before and after labor, do more harm than good. Too frequent examination should also be avoided.

Undoubtedly the use of rubber gloves, or at least of rubber finger cots, will still further reduce the danger of contagion, and their use should become universal.

Now, a few words in regard to some points in diagnosis and treatment. From what has been said, it will be understood that the recognition of the kind of infection is of the utmost importance. If we have to do simply with saprophytes, the treatment will be one thing; if we have streptococci, it will be another. And so with the different germs all along the line.

If there be a mixed infection, then the treatment must be influenced by this fact. Saprophytes and streptococci together in the uterus, might indicate two separate modes of treatment, both to be carried on at the same time.

An accurate diagnosis of every case is possible, especially in the large cities where the city maintains a bacteriological laboratory. The use of Döderlein's tubes will render it possible to have a careful bacteriological investigation made in every case. Whitridge Williams recommends the following plan:

Take a Döderlein tube which consists of a glass-tube 20 to 25 cm. in length and 3 to 4 mm. in diameter, with a slight bend at one end so as to conform to the anteflexed condition of the uterus. It is sterilised either by dry heat or steam, and is then ready for introduction. In practice the most convenient method for sterilising the tube and enabling us to carry it with us in a sterile condition is to place it in a long test-tube of thick glass, which contains at its lower extremity a small amount of cotton, and whose upper end is filled by a cotton plug, just as one closes the ordinary culture tubes which are employed in bacteriology. The lochial tube is then sterilised within the test-tube, and may thus be carried from place to place without fear of contamination.

When we wish to make cultures from the uterus our hands and the external genitalia should be thoroughly disinfected, the patient placed in Sims's position, and a sterilised Sims's or Simon's speculum introduced so as to retract the posterior vaginal wall, then the cervix caught with a sterile volsellum forceps and brought down to the vulva. The vaginal portion of the cervix is then carefully cleansed with a bit of sterilised cotton, and the sterile lochia tube is removed from its tube and introduced into the uterus as high up as it will go, care being taken to avoid touching the external genitals in the operation. To the end of the tube which protrudes from the vulva, a syringe, which draws well, is attached by means of a rubber tube. Suction is made, whereby a certain amount of the uterine contents is drawn up into the tube. The tube is then removed and its ends sealed with sealing-wax, when it can be carried to the laboratory without fear of contamination. On reaching the laboratory it is broken in its middle portion and culture taken from its contents, which we know represent the uncontaminated lochia from the upper part of the uterus.

Having accomplished the first step, we are now in position to act intelligently. If it be a simple endometritis with sapremia, and the condition is due to saprophytes, a thorough cleansing of the uterine cavity should be undertaken. The introduction of the carefully sterilised index finger will tell whether there is any roughness within the uterus, or any bits of placenta or membrane to be removed. If there are, then the curette should be used, with the abortion forceps, so as to make sure that every bit of placenta or membrane is taken out. Then the uterine cavity should be thoroughly irrigated with a mild antiseptic solution, and afterward packed with iodoform gauze.

If the bacteriological examination shows that the endometritis is due to streptococcus or staphylococcus infection, then very little will be accomplished by washing out the uterus, and the indications for the curette are done away with. It must be remembered that the streptococcus travels by the lymphatics. Observations show that it travels 2 cm. in the space of six hours. If, therefore, the disease is twelve hours old, the germ has got beyond the possibility of reaching it with the curette. Antiseptic injections are also perfectly useless, as they probably do not penetrate a single mm. into the tissues. All hopes of intrauterine treatment in streptococcus infection must be abandoned. Curetting in these cases not only does no good, but may do harm, as the reaction zone, which may possibly have grown up in the uterine tissue to resist the progress of the germs, will thus be scraped away and new tissue laid open to invasion. This reaction zone, however, plays a very small and unimportant part in the case of streptococcus invasion, as the progress of the germs is too rapid to allow of its formation.

If the infection be a late one and due to the gonococcus, as this germ travels by surfaces it may possibly be reached before it has gone too far. After it gets into the tubes there is very little chance of stopping its progress; but while within the cervix or the endometrium it may perhaps be stopped. Unquestionably, the use of the new silver compounds is the most rational method of treatment. The uterus can be washed out with a 2 per cent. solution of protargol, and the cavity packed with gauze smeared with Credé's ointment. Washing the uterus with the peroxide may also be useful. As a rule, however, this disease is very insidious, and we will hardly suspect its presence until it has gotten into the tubes or into the peritoneum. It does not often kill, the tendancy being rather to become chronic and to remain limited to the body of the uterus and tubes. The use of unguentum Credé rubbed into the skin would seem to offer prospect of relief. I have myself seen the most remarkable effects from it when used in a gonorrheal arthritis. If the tubes become infected and distended, they can be opened and drained by the cul-de-sac operation, which I shall mention later.

If the streptococcus makes its way so rapidly into the tissues as to be beyond the reach of the curette or the douche, is there any way by which its ravages can be stopped before the entire system is poisoned? A few years ago, Dr. Henrotin, of Chicago, proposed to attack it further on in its course. It is found that when the germ has penetrated the uterus, it accumulates upon the peritoneal surfaces, setting up a peritonitis. A large amount of fibrinous and serous exudate is quickly thrown out, which accumulates in the pelvis and becomes additional culture ground for the germs, which may soon set up a general peritonitis. Henrotin proposed to open the posterior fornix of the vagina into Douglas's cul-de-sac, and to drain away this fluid, at the same time thoroughly exploring with the finger, and then to pack the cul-de-sac with iodoform gauze, thus keeping up the drainage and introducing an efficient antiseptic. This plan has been followed in a number of cases, and is very strongly advocated by W. R. Pryor, who goes so far as to open the cul-de-sac in every case of puerperal infection in which he curettes. If there be a mixed infection—saprophytes and streptococci together, for instance—then Pryor's plan would seem to be a good one.

This operation may also be extended to the opening of recent accumulations of pus within the tubes or broad ligaments. I have opened recently formed pus-tubes in this way with eminently satisfactory results. The opening of the cul-de-sac is a very simple operation; little or no risk is run, and if nothing is found, the wound heals in a very short time. It seems to me to be one of the most important advances, for diagnosis and for treatment, and admits of application to many other than puerperal cases.

Another important question which is now before the profession is as to the propriety of removing the uterus where it is the seat of extensive infection. If it be riddled with abscesses, as is sometimes the case, that would seem to be the only means of effecting a cure. Authorities differ very much as to the propriety of hysterectomy for puerperal infection. It does not seem yet to be a settled mode of procedure, and the results in general are not very encouraging. Still a few operators have reported favorable statistics, some lives undoubtedly having been saved by this operation. The uterus should be taken out by the vagina, if at all, and the recent distention of the vagina and perineum, with the general relaxation of the parts, makes the operation comparatively easy.

I have thus passed in review some of the principal points in which advances have been made in the study and treatment of puerperal infection. It seems to me that there is great need of reform in this matter in Buffalo. While many practitioners do not see the disease at all, those who do a consulting practice will tell you that it is by no means so rare as is generally supposed. The reports of the Board of Health show that the disease is not by any means unknown; and these results are probably very unreliable, as physicians are apt not to report the true nature of the case when they lose a woman from puerperal infection. I have myself known of cases being reported as typhoid fever, pneumonia and the like.

I have chosen this subject for my address, in order that attention may again be attracted to it. I sincerely trust that in the near future the disease will be treated more scientifically and rationally than it has been in the past. The routine of curetting and douching every case should be given up, and these agencies only employed where the nature of the disease would seem to show that good may be accomplished. Nothing can be done by attacking a disease process which has passed the point of attack. It must be followed up and reached in its deeper lurking places, if any good is to be accomplished. Much remains to be done. The relations of certain bacteria to puerperal infection are not yet fully understood. Until this has been carefully worked out, it will be impossible to lay down a thoroughly rational plan of treatment where these little understood germs are the cause. Mixed infection is also a point demanding more knowledge. The whole subject is certainly worthy of the strongest effort, and one which may well occupy the brightest minds in the profession. There is no disease more important, no disease the study of which offers greater opportunities for success. Prophylaxis, however, must always be the great aim and object; and in the execution of this every practitioner must make himself an adept.

I cannot do better than to close by a quotation from Oliver Wendell Holmes. These sentences are among the best things in medical literature, and he must indeed be hardhearted who cannot yield himself to their influence:

I have no wish to express any harsh feeling with regard to the painful subject which has come before us. If there are any so far excited by the story of these dreadful events that they ask for some word of indignant remonstrance to show that science does not turn the hearts of its followers into ice or stone, let me remind them that such words have been uttered by those who speak with an authority I could not claim. It is as a lesson rather than as a reproach that I call up the memory of these irreparable errors and wrongs. No tongue can tell the heart-breaking calamity they have caused; they have closed the eyes just opened upon a new world of love and happiness; they have bowed the strength of manhood into the dust; they have cast the helplessness of infancy into the stranger's arms, or bequeathed it, with less cruelty, the death of its dying parent. There is no tone deep enough for regret, and no voice loud enough for warning. The woman about to become a mother, or with her newborn infant upon her bosom, should be the object of trembling care and sympathy wherever she bears her tender burden, or stretches her aching limbs. The very outcast of the streets has pity upon her sister in degradation, when the seal of promised maternity is impressed upon her. The remorseless vengeance of the law brought down upon its victim by a machinery as sure as destiny, is arrested in its fall at a word which reveals her transient claim for mercy. The solemn prayer of the liturgy singles out her sorrows from the multiplied trials of life, to plead for her in the hour of peril. God forbid that any member of the profession to which she trusts her life, doubly precious at that eventful period, should hazard it negligently, unadvisedly, or selfishly!

37 Allen Street.

